# Supplementary material for: Engineering a Phosphoketolase Pathway to Supplement Cytosolic Acetyl-CoA in Aspergillus niger Enables a Significant Increase in Citric Acid Production
Source: J Fungi (Basel). 2023 Apr 23;9(5):504. doi: 10.3390/jof9050504 (PMC10219267; doi:10.3390/jof9050504)
Supplement: Supplementary file 1 [file jof-09-00504-s001.zip › jof-2237924-supplementary.pdf]

Supplementary Materials

Engineering a phosphoketolase pathway to supplement cytoplasmic acetyl-CoA in *Aspergillus niger* enables a significant increase of citric acid production

Authors:

Jiao Liu[1,2], Shanshan Zhang[1], Wenhao Li[1], Guanyi Wang[1], Zhoujie Xie[1,2], Wei Cao[1,2], Weixia Gao[1,2], Hao Liu[1,2,3*]

Affiliation:

[1]MOE Key Laboratory of Industrial Fermentation Microbiology, College of Biotechnology, Tianjin University of Science & Technology, Tianjin 300457, China.

[2]Tianjin Engineering Research Center of Microbial Metabolism and Fermentation Process Control, Tianjin University of Science & Technology, Tianjin 300457, China.

[3] National Technology Innovation Center of Synthetic Biology, 300308, Tianjin, P. R. China.

*** Corresponding Authors:**

Hao Liu

Phone: (86)-22-60600810. Fax: (86)-22-60602298. Email: liuhao@tust.edu.cn

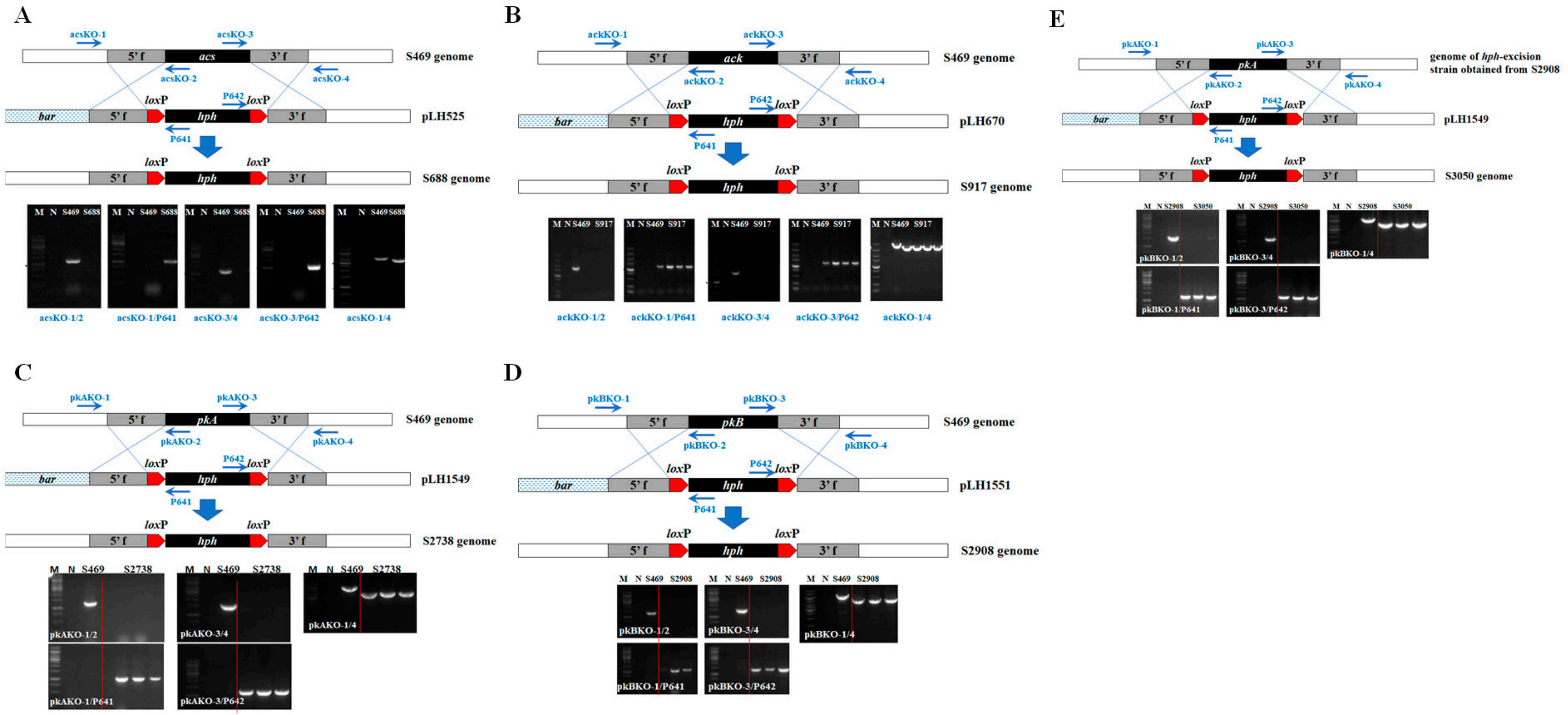

Figure S1. Schematic of homologous recombination and the electrophoretic analyses of the verification PCR products for single deletion of *acs*, *ack*, *pkA*, *pkB*(A-D) and double deletion *pkA* and *pkB*(E).

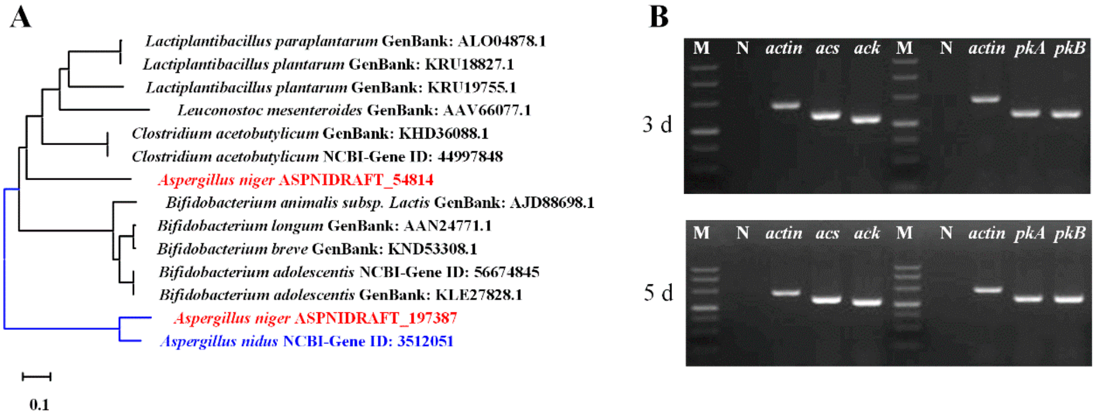

Figure S2. Neighbor-joning phylogenetic tree of PKs from different microorganisms, including bacteria, *A. nidulans* and *A. niger*, established using MEGA software Version 10.1.7. (A) and the 3 and 5 d RT-PCR analysis of *acs*, *ack*, *pkA* and *pkB* of *A. niger* S469 during the shake flask citrate fermentation at 28 °C with a shaking speed of 200 rpm.

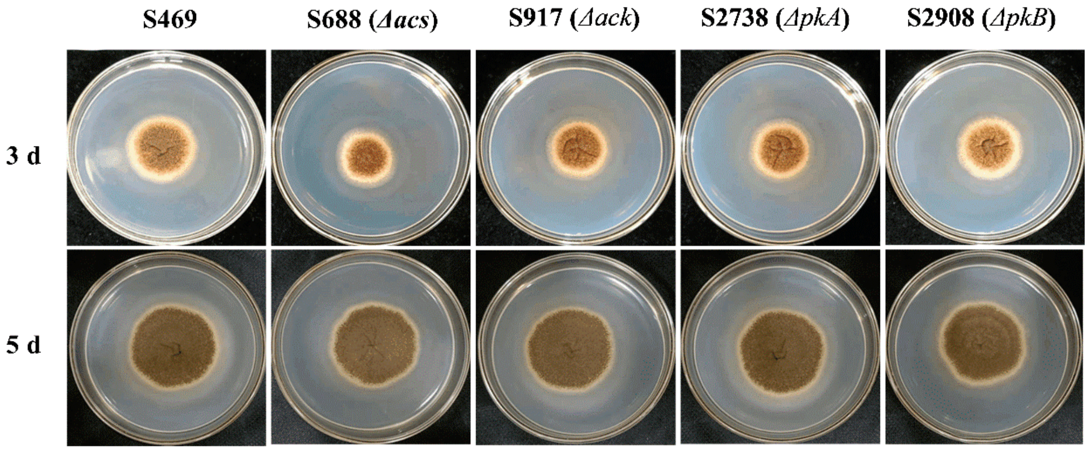

Figure S3. Morphology of *A. niger* S469, the Δ*acs* mutant S688, the Δ*ack* mutant S917, the Δ*pkA* mutant S2738 and the Δ*pkB* mutant S2908 grown on PDA plate at 28°C for 3 d and 5 d.

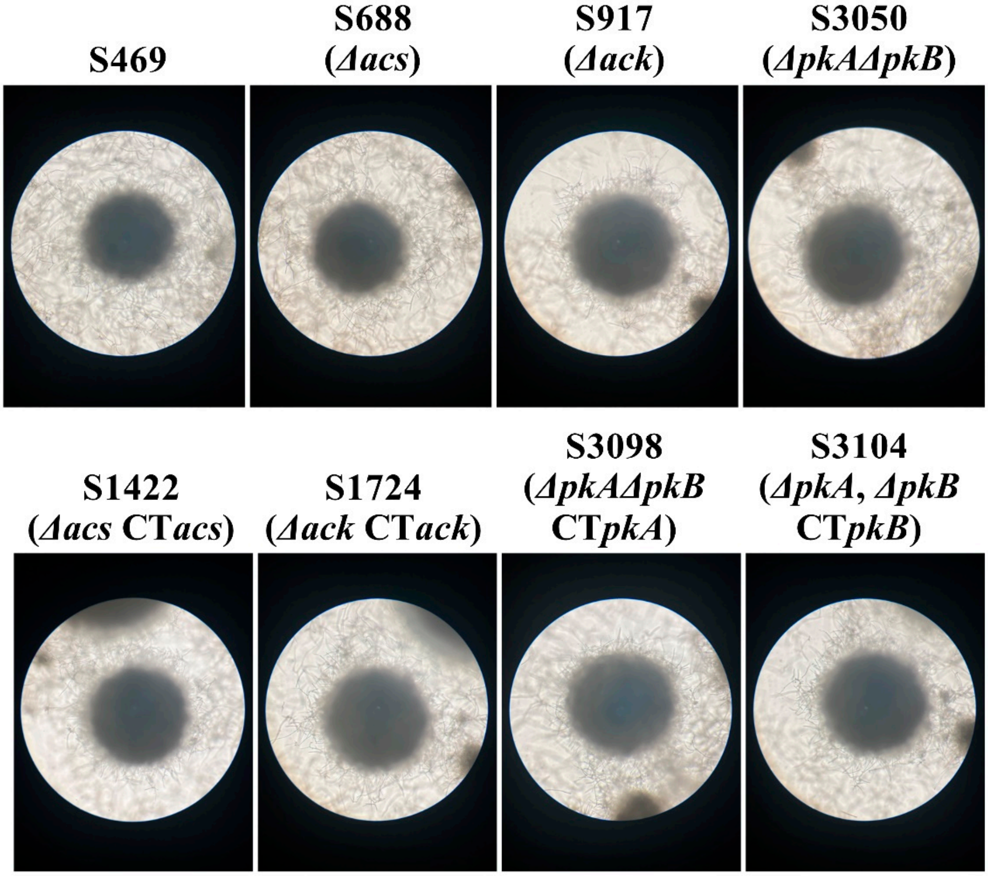

Figure S4. Morphology of *A. niger* S469, the Δ*acs* mutant S688, the Δ*ack* mutant S917, the Δ*pkA*Δ*pkB* mutant S3050, the Δ*acs* CT*acs* mutant S1422, the Δ*ack* CT*ack* mutant S1724, the Δ*pkA*Δ*pkB* CT*pkA* mutant S3098 and the Δ*pkA*Δ*pkB* CT*pkB* mutant S23104 at a 3-day citrate fermentation in a shake flask at 28 °C at 200 rpm. The morphology of mycelial pellets was observed under a microscope magnified by 40 times. CT: complementation of ack driven by the *gpdA* promoter at the *amyA* locus.

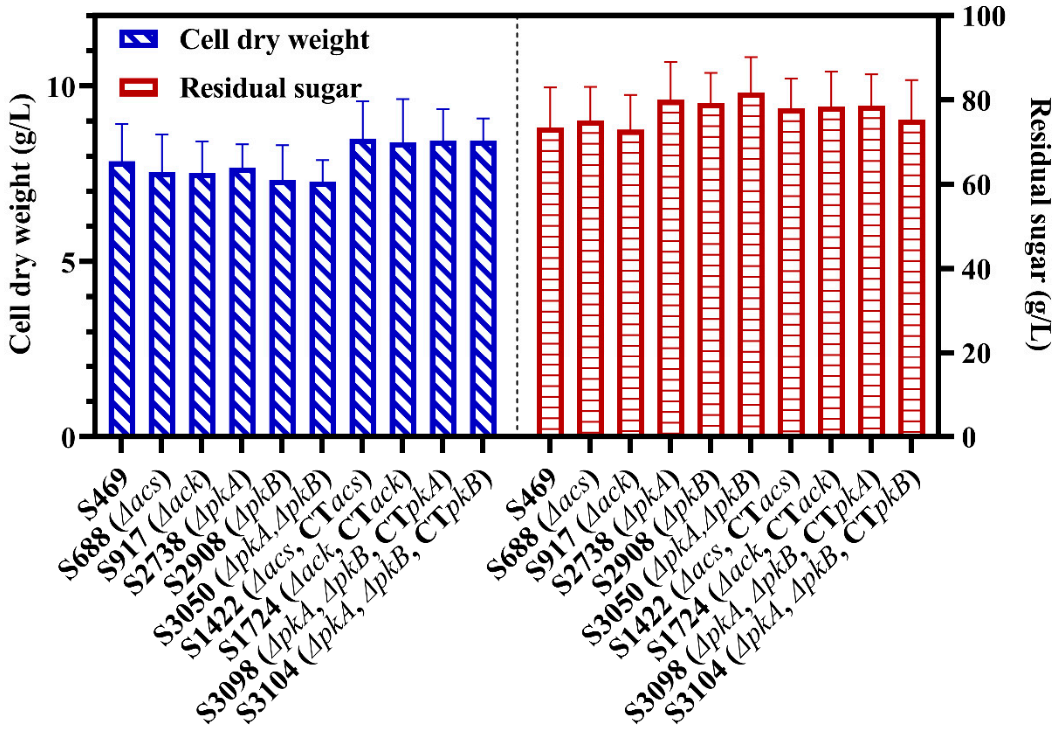

Figure S5. The cell dry weights and residual sugars of *A. niger* S469, the Δ*acs* mutant S688, the Δ*ack* mutant S917, the Δ*pkA* mutant S2738, the Δ*pkB* mutant S2908, the Δ*pkA*Δ*pkB* mutant S3050, the Δ*acs* CT*acs* mutant S1422, the Δ*ack* CT*ack* mutant S1724, the Δ*pkA*Δ*pkB* CT*pkA* mutant S3098 and the Δ*pkA*Δ*pkB* CT*pkB* mutant S23104 at a 3-day citrate fermentation in a shake flask at 28 °C at 200 rpm. CT: complementation of ack driven by the *gpdA* promoter at the *amyA* locus.

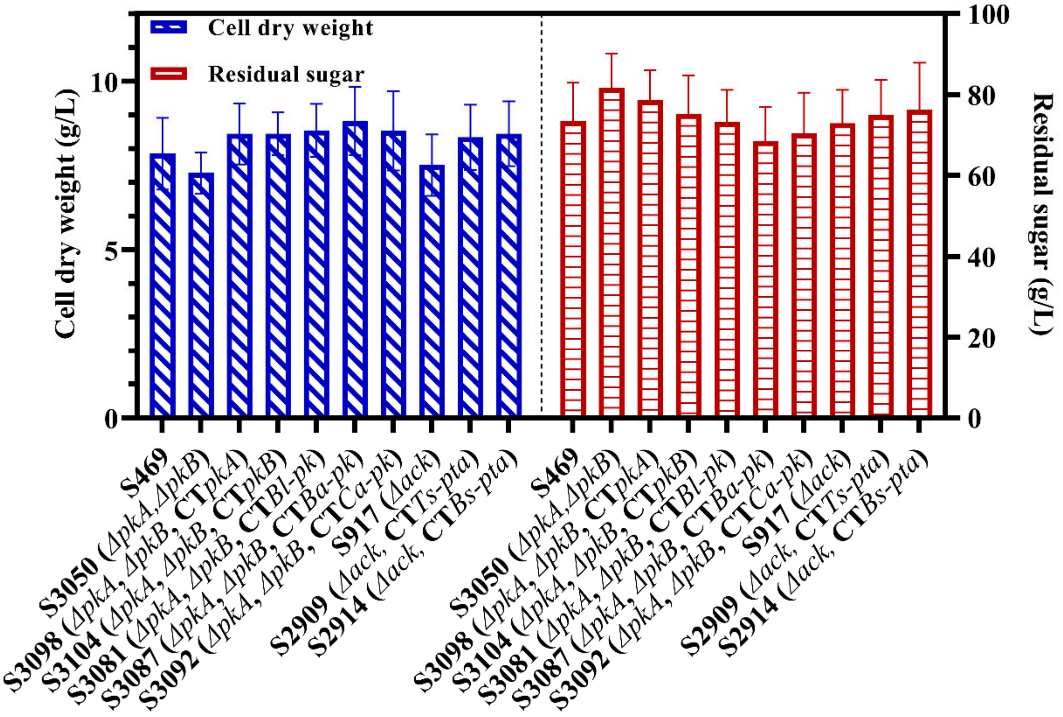

Figure S6. The cell dry weights and residual sugars of *A. niger* S469, the Δ*pkA*Δ*pkB* mutant S3050, the Δ*pkA*Δ*pkB* CT*pkA* mutant S3098, the Δ*pkA*Δ*pkB* CT*pkB* mutant S23104, the Δ*pkA*Δ*pkB* CT*Bl-pkB* mutant S3081, the Δ*pkA*Δ*pkB* CT*Ba-pkB* mutant S3087, the Δ*pkA*Δ*pkB* CT*Ca-pkB* mutant S3092, the Δ*ack* mutant S917, the Δ*ack* CT*Ts-pta* mutant S2909 and the Δ*ack* CT*Bs-pta* mutant S2914 at a 3-day citrate fermentation in a shake flask at 28 °C at 200 rpm. CT: complementation of ack driven by the *gpdA* promoter at the *amyA* locus.

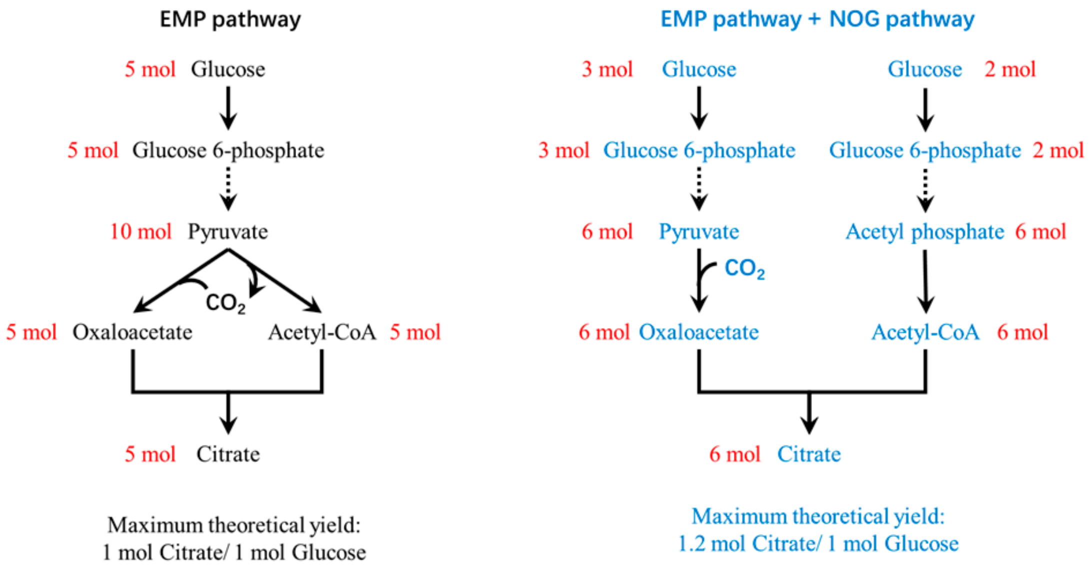

Figure S7. Maximum theoretical yield of citrate from glucose through EMP pathway was 1 mol citrate/ 1 mol glucose, and that through combined EMP pathway and NOG pathway reached to 1.2 mol citrate/ 1 mol glucose.

Table S1. Primers used in this study

Primers	Sequences (5´→3´)
Primers used for amplification of *acs*-5'f sequence	
acsU-F	CAGGACTGAAGGCCTGAATTCCATGGTGGGAAGTAGACC
acsU-R	CGAAGTTATGGATCCGAGCTCGAAATTCCATTCGTAGCG
Primers used for amplification of *acs*-3'f sequence	
acsD-F	GCTATACGAAGTTATTCTAGATGAACCGAATCCAAGACG
acsD-R	ACGACGGCCAGTGCCAAGCTTGGGAAATAATGACGAGGTG
Primers used for confirmation of *acs* deletion	
acsKO-1	CCTCCCACCCTGTTCATT
acsKO-2	TGTCGGGTGCTTCTGGTG
acsKO-3	GCCTGGGTGACACTTCTA
acsKO-4	AGGGAGTCATTCGGTTCG
P641	CAATATCAGTTAACGTCGAC
P642	GGAACCAGTTAACGTCGAAT
Primers used for amplification of *ack*-5'f sequence	
ackU-F	GCTCCGTAACACCCAGAATTCCCGCATTGATATGGCTTGA
ackU-R	ATTATACGAAGTTATGGATCCGTGGGTTGATGCTTGTTGGT
Primers used for amplification of *ack*-3'f sequence	
ackD-F	GCTATACGAAGTTATTCTAGACCAAGAACCGTCCACCAA
ackD-R	GCCAAGCTTGCATGCCTGCAGCTGCCACATTGATGCTCC
Primers used for confirmation of *ack* deletion	
ackKO-1	CCATAACCCGTGAAGAGG
ackKO-2	TGAAGGCATCTTGAGGAGTA
ackKO-3	CACATCACCCGACAACAA
ackKO-4	AGATGCTGGATTCCTTGT
P641	CAATATCAGTTAACGTCGAC
P642	GGAACCAGTTAACGTCGAAT
Primers used for amplification of *pkA*-5'f sequence	
pkAU-F	CCCAGAATTCAATTCGAGCTCCAGCAGGTTTGTTCAGGAC
pkAU-R	ATTATACGAAGTTATGGATCCAAGTATCAGGGTCCGTTCG
Primers used for amplification of *pkA*-3'f sequence	
pkAD-F	GCTATACGAAGTTATTCTAGAAGATTTTGACACCTAGGTTCTC
pkAD-R	TGCCTGCAGGGGCCCACTAGTCCATTACTCTATTCTCGTCCG
Primers used for confirmation of *pkA* deletion	
pkAKO-1	CGACGATAAGACTGTGAGTCTC
pkAKO-2	GTGAAGGCTCAGCCTTG
pkAKO-3	CAACATCGTGGAGACACGC
pkAKO-4	CAGGCTGAGCGCTAATCG
P641	CAATATCAGTTAACGTCGAC
P642	GGAACCAGTTAACGTCGAAT
Primers used for amplification of *pkB*-5'f sequence	
pkBU-F	CCCAGAATTCAATTCGAGCTCCCACATCTGTATCCTTTGCAC
pkBU-R	ATTATACGAAGTTATGGATCCACTTGGTAAAATGCCGTTAGTTG

Primers used for amplification of pkB-3’f sequence	
pkBD-F	GCTATACGAAGTTATTCTAGACTACTAGGAAAGTCGCCCC
pkBD-R	TGCCTGCAGGGGCCCACTAGTAGTTGGTTGTCATGACTGTGG
Primers used for confirmation of *pkB* deletion	
pkBKO-1	GCTCTTCTCTGTGTCTCTTGC
pkBKO-2	TACATGCTGGCGCGAAAG
pkBKO-3	ATGCAGTTATTGGCCAAGC
pkBKO-4	TGCTGGGGAAGATGACGG
P641	CAATATCAGTTAACGTCGAC
P642	GGAACCAGTTAACGTCGAAT
Primers used for coding gene of *acs*	
acs-F	ATAGACACATCTAAACAATGGAATTCATGGCCGACGGCAGTGTT ATG
acs-R	CAGTAACGTTAAGTGGATCCCTGCAGTTATTTGCCACGGGCAGC GTGC
Primers used for coding gene of *ack*	
ack-F	ATCAATCATCCGTCAAGATGGGAATTCATGGCGCGCAAATCCAT CCTC
ack-R	GTGGATCCAGATCTCTGCAGGGTACCCTACGGCCTCTCATACTG
Primers used for coding gene of *Bl-pk*	
Bl-pk-L	CACATCTAAACAATGGAATTCATGACCTCCCCCGTCATC
Bl-PK-R	TCCCTGCAGGGTACCGAGCTCTCACTCGTTGTCGCCGG
Primers used for coding gene of *Ba-pk*	
Ba-PK-L	CACATCTAAACAATGGAATTCATGACCTCCCCCGTCATC
Ba-PK-R	TCCCTGCAGGGTACCGAGCTCTCACTCGTTGTCGCCAGC
Primers used for coding gene of *Ca-pk*	
Ca-pk-L	AGACACATCTAAACAATGCAGTCCATCATCGGC
Ca-pk-R	AGTGGATCCCTGCAGGGTACCGAGCTCTCAGACGTGCCAC
Primers used for coding gene of *pkA*	
pkA-L	CACATCTAAACAATGCCTGGAGAGGTCATCGACAG
pkA-R	TCCCTGCAGGGTACCGAGCTCTTATTCAAAGGAGGGCATATC
Primers used for coding gene of *pkB*	
pkB-L	CACATCTAAACAATGCCATCGGATTCGAATGATC
pkB-R	TCCCTGCAGGGTACCGAGCTCTCAGACGCCCTCCCCC
Primers used for coding gene of *Ts-pta*	
Ts-pta-L	CACATCTAAACAATGGAATTCATGTCCATCATTCAGAACATC
Ts-pta-R	TCCCTGCAGGGTACCGAGCTCTCACTGAGCTTGGACGG
Primers used for coding gene of *Bs-pta*	
Bs-pta-L	CACATCTAAACAATGGCCGACCTGTTCTCCACCGTC
Bs-pta-R	TCCCTGCAGGGTACCGAGCTCTCACAGGGCTTGGGCGGC
Primers used for gene expression cassetes	
P924-454-L	GGGAGAATTCAATTCGAGCTCGAGACTAGTGGACTAACATTATT CC

P924-454-R	ATTATACGAAGTTATGGATCCGTCTAGAAAGAAGGATTACCTCT AAAC
Primers used for coding gene of *Ca-pk* under control of *PgdhA*	
PgdhACapk-L	CTCCCCTTCAGAATGGAATTCCAGTCCATCATCGGCAAG
PgdhACapk-R	TCAGTAACGTTAAGTGGATCCTCAGACGTGCCACTGCC
Primers used for gene expression cassete of *Ca-pk* under control of *PgdhA*	
P1804-L	GAGGTAATCCTTCTTGAGTAAGGATCTCCGGGG
P1804-R	TAGGGCCCCCCGGGGTCTAGAAAGAAGGATTACCTC
Primers used for verification of *hph*-excision strain	
hph-F	CCGCGACGTCTGTCGAGAAGTTTC
hph-R	CCTTTGCCCTCGGACGAGTGC
Primers used for RT-PCR of *acs*	
RTPCR-acs-F	CCACTGCCCTGCGACTTC
RTPCR-acs-R	GAACCCGTCTCAGTCTGCCAG
Primers used for RT-PCR of *ack*	
RTPCR-ack-F	CCACTACACCAACGATGCAGG
RTPCR-ack-R	GCCAGTTTATGTTCCCGTGTCG
Primers used for RT-PCR of *pkA*	
RTPCR-pkA-F	CGACGCAGATCTCCATAGCTC
RTPCR-pkA-R	GCCATTTCTGCAGAGCCTG
Primers used for RT-PCR of *pkB*	
RTPCR-pkB-F	CTGGCCCATGATCATACTGCG
RTPCR-pkB-R	GGCCGTCGTGAGTGAAGAGC
Primers used for RT-PCR of *actin*	
RTPCR-actin-F	TCCTCACCCTCAGATACCC
RTPCR-actin-R	CACCGTCACCAGAGTCCA

Supplementary Material. Sequence of the plasmid pLH1080

AGCTTGGCACTGGCCGTCGTTTTACAACGTCGTGACTGGGAAAACCCTGGCGTTACCCAACTTAATCGCCTTGCAGCACATC
CCCCTTTCGCCAGCTGGCGTAATAGCGAAGAGGCCCGCACCGATCGCCCTTCCCAACAGTTGCGCAGCCTGAATGGCGAAT
GCTAGAGCAGCTTGAGCTTGGATCAGATTGTCGTTTCCCGCCTTCAGTTTAAACTATCAGTGTTTGACAGGATATATTGGCGG
GTAAACCTAAGAGAAAAGAGCGTTTATTAGAATAACGGATATTTAAAAGGGCGTGAAAAGGTTTATCCGTTCGTCCATTTGTA
TGTGCATGCCAACCACAGGGTTCCCCTCGGGATCAAAGTACTTTGATCCAACCCCTCCGCTGCTATAGTGCAGTCGGCTTCT
GACGTTCAGTGCAGCCGTCTTCTGAAAACGACATGTCGCACAAGTCCTAAGTTACGCGACAGGCTGCCGCCCTGCCCTTTTC
CTGGCGTTTTCTTGTCGCGTGTTTTAGTCGCATAAAGTAGAATACTTGCGACTAGAACCGGAGACATTACGCCATGAACAAG
AGCGCCGCCGCTGGCCTGCTGGGCTATGCCCGCGTCAGCACCGACGACCAGGACTTGACCAACCAACGGGCCGAACTGCA
CGCGGCCGGCTGCACCAAGCTGTTTTCCGAGAAGATCACCGGCACCAGGCGCGACCGCCCGGAGCTGGCCAGGATGCTTG
ACCACCTACGCCCTGGCGACGTTGTGACAGTGACCAGGCTAGACCGCCTGGCCCGCAGCACCCGCGACCTACTGGACATTG
CCGAGCGCATCCAGGAGGCCGGCGCGGGCCTGCGTAGCCTGGCAGAGCCGTGGGCCGACACCACCACGCCGGCCGGCCGC
ATGGTGTTGACCGTGTTCGCCGGCATTGCCGAGTTCGAGCGTTCCCTAATCATCGACCGCACCCGGAGCGGGCGCGAGGCC
GCCAAGGCCCGAGGCGTGAAGTTTGGCCCCCGCCCTACCCTCACCCCGGCACAGATCGCGCACGCCCGCGAGCTGATCGAC
CAGGAAGGCCGCACCGTGAAAGAGGCGGCTGCACTGCTTGGCGTGCATCGCTCGACCCTGTACCGCGCACTTGAGCGCAG
CGAGGAAGTGACGCCCACCGAGGCCAGGCGGCGCGGTGCCTTCCGTGAGGACGCATTGACCGAGGCCGACGCCCTGGCGG
CCGCCGAGAATGAACGCCAAGAGGAACAAGCATGAAACCGCACCAGGACGGCCAGGACGAACCGTTTTTCATTACCGAAG
AGATCGAGGCGGAGATGATCGCGGCCGGGTACGTGTTCGAGCCGCCCGCGCACGTCTCAACCGTGCGGCTGCATGAAATCC
TGGCCGGTTTGTCTGATGCCAAGCTGGCGGCCTGGCCGGCCAGCTTGGCCGCTGAAGAAACCGAGCGCCGCCGTCTAAAAA
GGTGATGTGTATTTGAGTAAAACAGCTTGCGTCATGCGGTCGCTGCGTATATGATGCGATGAGTAAATAAACAAATACGCAAG
GGGAACGCATGAAGGTTATCGCTGTACTTAACCAGAAAGGCGGGTCAGGCAAGACGACCATCGCAACCCATCTAGCCCGCG

CCCTGCAACTCGCCGGGGCCGATGTTCTGTTAGTCGATTCCGATCCCCAGGGCAGTGCCCGCGATTGGGCGGCCGTGCGGG
AAGATCAACCGCTAACCGTTGTCGGCATCGACCGCCCGACGATTGACCGCGACGTGAAGGCCATCGGCCGGCGCGACTTCG
TAGTGATCGACGGAGCGCCCCAGGCGGCGGACTTGGCTGTGTCCGCGATCAAGGCAGCCGACTTCGTGCTGATTCCGGTGC
AGCCAAGCCCTTACGACATATGGGCCACCGCCGACCTGGTGGAGCTGGTTAAGCAGCGCATTGAGGTCACGGATGGAAGGC
TACAAGCGGCCTTTGTCGTGTCGCGGGCGATCAAAGGCACGCGCATCGGCGGTGAGGTTGCCGAGGCGCTGGCCGGGTACG
AGCTGCCCATTCTTGAGTCCCGTATCACGCAGCGCGTGAGCTACCCAGGCACTGCCGCCGCCGGCACAACCGTTCTTGAATC
AGAACCCGAGGGCGACGCTGCCCGCGAGGTCCAGGCGCTGGCCGCTGAAATTAAATCAAAACTCATTTGAGTTAATGAGGT
AAAGAGAAAATGAGCAAAAGCACAAACACGCTAAGTGCCGGCCGTCCGAGCGCACGCAGCAGCAAGGCTGCAACGTTGG
CCAGCCTGGCAGACACGCCAGCCATGAAGCGGGTCAACTTTCAGTTGCCGGCGGAGGATCACACCAAGCTGAAGATGTAC
GCGGTACGCCAAGGCAAGACCATTACCGAGCTGCTATCTGAATACATCGCGCAGCTACCAGAGTAAATGAGCAAATGAATAA
ATGAGTAGATGAATTTTAGCGGCTAAAGGAGGCGGCATGGAAAATCAAGAACAACCAGGCACCGACGCCGTGGAATGCCCC
ATGTGTGGAGGAACGGGCGGTTGGCCAGGCGTAAGCGGCTGGGTTGTCTGCCGGCCCTGCAATGGCACTGGAACCCCCAA
GCCCGAGGAATCGGCGTGACGGTCGCAAACCATCCGGCCCGGTACAAATCGGCGCGGCGCTGGGTGATGACCTGGTGGAG
AAGTTGAAGGCCGCGCAGGCCGCCCAGCGGCAACGCATCGAGGCAGAAGCACGCCCCGGTGAATCGTGGCAAGCGGCCG
CTGATCGAATCCGCAAAGAATCCCGGCAACCGCCGGCAGCCGGTGCGCCGTCGATTAGGAAGCCGCCCAAGGGCGACGAG
CAACCAGATTTTTTCGTTCCGATGCTCTATGACGTGGGCACCCGCGATAGTCGCAGCATCATGGACGTGGCCGTTTTCCGTCT
GTCGAAGCGTGACCGACGAGCTGGCGAGGTGATCCGCTACGAGCTTCCAGACGGGCACGTAGAGGTTTCCGCAGGGCCGG
CCGGCATGGCCAGTGTGTGGGATTACGACCTGGTACTGATGGCGGTTTCCCATCTAACCGAATCCATGAACCGATACCGGGA
AGGGAAGGGAGACAAGCCCGGCCGCGTGTTCCGTCCACACGTTGCGGACGTACTCAAGTTCTGCCGGCGAGCCGATGGCG
GAAAGCAGAAAGACGACCTGGTAGAAACCTGCATTCGGTTAAACACCACGCACGTTGCCATGCAGCGTACGAAGAAGGCC
AAGAACGGCCGCCTGGTGACGGTATCCGAGGGTGAAGCCTTGATTAGCCGCTACAAGATCGTAAAGAGCGAAACCGGGCG
GCCGGAGTACATCGAGATCGAGCTAGCTGATTGGATGTACCGCGAGATCACAGAAGGCAAGAACCCGGACGTGCTGACGGT
TCACCCCGATTACTTTTTGATCGATCCCGGCATCGGCCGTTTTCTCTACCGCCTGGCACGCCGCGCCGCAGGCAAGGCAGAA
GCCAGATGGTTGTTCAAGACGATCTACGAACGCAGTGGCAGCGCCGGAGAGTTCAAGAAGTTCTGTTTCACCGTGCGCAAG
CTGATCGGGTCAAATGACCTGCCGGAGTACGATTTGAAGGAGGAGGCGGGGCAGGCTGGCCCGATCCTAGTCATGCGCTAC
CGCAACCTGATCGAGGGCGAAGCATCCGCCGGTTCCTAATGTACGGAGCAGATGCTAGGGCAAATTGCCCTAGCAGGGGAA
AAAGGTCGAAAAGGTCTCTTTCCTGTGGATAGCACGTACATTGGGAACCCAAAGCCGTACATTGGGAACCGGAACCCGTAC
ATTGGGAACCCAAAGCCGTACATTGGGAACCGGTCACACATGTAAGTGACTGATATAAAAGAGAAAAAAGGCGATTTTTCC
GCCTAAAACTCTTTAAAACTTATTAAAACTCTTAAAACCCGCCTGGCCTGTGCATAACTGTCTGGCCAGCGCACAGCCGAAG
AGCTGCAAAAAGCGCCTACCCTTCGGTCGCTGCGCTCCCTACGCCCCGCCGCTTCGCGTCGGCCTATCGCGGCCGCTGGCCG
CTCAAAAATGGCTGGCCTACGGCCAGGCAATCTACCAGGGCGCGGACAAGCCGCGCCGTCGCCACTCGACCGCCGGCGCC
CACATCAAGGCACCCTGCCTCGCGCGTTTCGGTGATGACGGTGAAAACCTCTGACACATGCAGCTCCCGGAGACGGTCACA
GCTTGTCTGTAAGCGGATGCCGGGAGCAGACAAGCCCGTCAGGGCGCGTCAGCGGGTGTTGGCGGGTGTCGGGGCGCAGC
CATGACCCAGTCACGTAGCGATAGCGGAGTGTATACTGGCTTAACTATGCGGCATCAGAGCAGATTGTACTGAGAGTGCACC
ATATGCGGTGTGAAATACCGCACAGATGCGTAAGGAGAAAATACCGCATCAGGCGCTCTTCCGCTTCCTCGCTCACTGACTC
GCTGCGCTCGGTCGTTCGGCTGCGGCGAGCGGTATCAGCTCACTCAAAGGCGGTAATACGGTTATCCACAGAATCAGGGGAT
AACGCAGGAAAGAACATGTGAGCAAAAGGCCAGCAAAAGGCCAGGAACCGTAAAAAGGCCGCGTTGCTGGCGTTTTTCCA
TAGGCTCCGCCCCCCTGACGAGCATCACAAAAATCGACGCTCAAGTCAGAGGTGGCGAAACCCGACAGGACTATAAAGATA
CCAGGCGTTTCCCCCTGGAAGCTCCCTCGTGCGCTCTCCTGTTCCGACCCTGCCGCTTACCGGATACCTGTCCGCCTTTCTCC
CTTCGGGAAGCGTGGCGCTTTCTCATAGCTCACGCTGTAGGTATCTCAGTTCGGTGTAGGTCGTTCGCTCCAAGCTGGGCTG
TGTGCACGAACCCCCCGTTCAGCCCGACCGCTGCGCCTTATCCGGTAACTATCGTCTTGAGTCCAACCCGGTAAGACACGAC
TTATCGCCACTGGCAGCAGCCACTGGTAACAGGATTAGCAGAGCGAGGTATGTAGGCGGTGCTACAGAGTTCTTGAAGTGG
TGGCCTAACTACGGCTACACTAGAAGGACAGTATTTGGTATCTGCGCTCTGCTGAAGCCAGTTACCTTCGGAAAAAGAGTTG
GTAGCTCTTGATCCGGCAAACAAACCACCGCTGGTAGCGGTGGTTTTTTTGTTTGCAAGCAGCAGATTACGCGCAGAAAAA
AAGGATCTCAAGAAGATCCTTTGATCTTTTCTACGGGGTCTGACGCTCAGTGGAACGAAAACTCACGTTAAGGGATTTTGGT
CATGCATTCTAGGTACTAAAACAATTCATCCAGTAAAATATAATATTTTATTTTCTCCCAATCAGGCTTGATCCCCAGTAAGTCA
AAAAATAGCTCGACATACTGTTCTTCCCCGATATCCTCCCTGATCGACCGGACGCAGAAGGCAATGTCATACCACTTGTCCGC
CCTGCCGCTTCTCCCAAGATCAATAAAGCCACTTACTTTGCCATCTTTCACAAAGATGTTGCTGTCTCCCAGGTCGCCGTGGG
AAAAGACAAGTTCCTCTTCGGGCTTTTCCGTCTTTAAAAAATCATACAGCTCGCGCGGATCTTTAAATGGAGTGTCTTCTTCC
CAGTTTTCGCAATCCACATCGGCCAGATCGTTATTCAGTAAGTAATCCAATTCGGCTAAGCGGCTGTCTAAGCTATTCGTATAG
GGACAATCCGATATGTCGATGGAGTGAAAGAGCCTGATGCACTCCGCATACAGCTCGATAATCTTTTCAGGGCTTTGTTCATC
TTCATACTCTTCCGAGCAAAGGACGCCATCGGCCTCACTCATGAGCAGATTGCTCCAGCCATCATGCCGTTCAAAGTGCAGG
ACCTTTGGAACAGGCAGCTTTCCTTCCAGCCATAGCATCATGTCCTTTTCCCGTTCCACATCATAGGTGGTCCCTTTATACCGG
CTGTCCGTCATTTTTAAATATAGGTTTTCATTTTCTCCCACCAGCTTATATACCTTAGCAGGAGACATTCCTTCCGTATCTTTTA
CGCAGCGGTATTTTTCGATCAGTTTTTTCAATTCCGGTGATATTCTCATTTTAGCCATTTATTATTTCCTTCCTCTTTTCTACAGT
ATTTAAAGATACCCCAAGAAGCTAATTATAACAAGACGAACTCCAATTCACTGTTCCTTGCATTCTAAAACCTTAAATACCAG
AAAACAGCTTTTTCAAAGTTGTTTTCAAAGTTGGCGTATAACATAGTATCGACGGAGCCGATTTTGAAACCGCGGTGATCAC
AGGCAGCAACGCTCTGTCATCGTTACAATCAACATGCTACCCTCCGCGAGATCATCCGTGTTTCAAACCCGGCAGCTTAGTT

GCCGTTCTTCCGAATAGCATCGGTAACATGAGCAAAGTCTGCCGCCTTACAACGGCTCTCCCGCTGACGCCGTCCCGGACTG
ATGGGCTGCCTGTATCGAGTGGTGATTTTGTGCCGAGCTGCCGGTCGGGGAGCTGTTGGCTGGCTGGTGGCAGGATATATTG
TGGTGTAAACAAATTGACGCTTAGACAACTTAATAACACATTGCGGACGTTTTTAATGTACTGAATTAACGCCGAATTAATTC
GGGGGATCTGGATTTTAGTACTGGATTTTGGTTTTAGGAATTAGAAATTTTATTGATAGAAGTATTTTACAAATACAAATACATA
CTAAGGGTTTCTTATATGCTCAACACATGAGCGAAACCCTATAGGAACCCTAATTCCCTTATCTGGGAACTACTCACACATTAT
TATGGAGAAACTCGAGACTAGTGAGTAAGGATCTCCGGGGAGGGGAAAGAGAGATAGACATTTGGGAAGGGGAAATAGGG
TGAAGATGAGGTTGTATTGGCTGAGTGCAGAGGATTTGACGATACGAGTAGGTAAAGGAAATCCGGACATCTGATAAGCACC
TCCCGTGCAGCGGGGGGAGCCAGGCCAATGGGAAGGGAGATGTCAGGTGATCCGCCAGTCTGCAGGATCTCTTGGCGTGC
AGGGCAAATGCGAGTTTCTCTCCAGATTTCAGCAGGTTCTAGATTGCGACGGCGTATTGCTTATCCTTAGTAGGACTCCCTAA
TGGATTCCGAGCAAGAAAAGACTGTTTGGCGTGTACCAATGGCTCATAGTACCAGCAAGAGAAGAATTTTCTCTCTCGCTTC
GAGAAAGCAATCAAAAAAAAATCCTATCCTACCCTACCCTACCCTAATACTTCCATTGCCACCCGATTCCTCCCGATAGTAGA
GCGGGCGACTGCCATTGGCGGGCGGGCCAGCGGATTCCCGCCGATAGATAACGGGCAGATTCTGTGACCTCAAACTATCGA
CTAACAGCCCGAACTTCGGCGCCACCGCCAAACCCGCCCCGGAAGCCGGCCTCATTTGCCGTTTGGGGCGTGCCAGGAAAT
GCCCGCCTGCAGCGGAGACTCCCTAGTGTGGTCTGTGTTGCCTGTGTCGTCTGTGTAGTATACTAGTTACTAGTCTACTACTG
TACAGTGGATGGCCTGAGGGGGGGACTTTATGTCCGACTCCGGCTGTTCTCCTCCCTCTATCCACTCTACCCTCTTCCCTCTC
TTCTGTCTTTCTCCCCGCTCTCGCCCCTCCCCTCCTCGAAAACATAAATCGGCCTTTCCCCCTCGCCATCTTCTTCTTCTTCTC
CCTCTCCTTTCTCTTTCTTCTTCAGACTACTTCTCTTTCTTTCATCTTTTCTCTATATTCCTGTTTTCCTAGATACCCCAGTTAAA
AAAGTTCTCTCAATCAATCCTCCCCTTCAGAATGGAATTCGAGCTCGGTACCCTGCAGGGATCCACTTAACGTTACTGAAATC
ATCAAACAGCTTGACGAATCTGGATATAAGATCGTTGGTGTCGATGTCAGCTCCGGAGTTGAGACAAATGGTGTTCAGGATC
TCGATAAGATACGTTCATTTGTCCAAGCAGCAAAGAGTGCCTTCTAGTGATTTAATAGCTCCATGTCAACAAGAATAAAACGC
GTTTTCGGGTTTACCTCTTCCAGATACAGCTCATCTGCAATGCATTAATGCATTGACTGCAACCTAGTAACGCCTTNCAGGCT
CCGGCGAAGAGAAGAATAGCTTAGCAGAGCTATTTTCATTTTCGGGAGACGAGATCAAGCAGATCAACGGTCGTCAAGAGA
CCTACGAGACTGAGGAATCCGCTCTTGGCTCCACGCGACTATATATTTGTCTCTAATTGTACTTTGACATGCTCCTCTTCTTTA
CTCTGATAGCTTGACTATGAAAATTCCGTCACCAGCNCCTGGGTTCGCAAAGATAATTGCATGTTTCTTCCTTGAACTCTCAA
GCCTACAGGACACACATTCATCGTAGGTATAAACCTCGAAATCANTTCCTACTAAGATGGTATACAATAGTAACCATGCATGG
TTGCCTAGTGAATGCTCCGTAACACCCAATACGCCGGCCGAAACTTTTTTACAACTCTCCTATGAGTCGTTTACCCAGAATGC
ACAGGTACACTTGTTTAGAGGTAATCCTTCTTTCTAGACCCGGGGGGCCCTACGTA**ATAACTTCGTATAATGTATGCTATAC**
GAAGTTATGTCGACGTTAACTGATATTGAAGGAGCATTTTTTGGGCTTGGCTGGAGCTAGTGGAGGTCAACAATGAATGCCT
ATTTTGGTTTAGTCGTCCAGGCGGTGAGCACAAAATTTGTGTCGTTTGACAAGATGGTTCATTTAGGCAACTGGTCAGATCA
GCCCCACTTGTAGCAGTAGCGGCGGCGCTCGAAGTGTGACTCTTATTAGCAGACAGGAACGAGGACATTATTATCATCTGCT
GCTTGGTGCACGATAACTTGGTGCGTTTGTCAAGCAAGGTAAGTGGACGACCCGGTCATACCTTCTTAAGTTCGCCCTTCCT
CCCTTTATTTCAGATTCAATCTGACTTACCTATTCTACCCAAGCATCCAAATGAAAAAGCCTGAACTCACCGCGACGTCTGTC
GAGAAGTTTCTGATCGAAAAGTTCGACAGCGTCTCCGACCTGATGCAGCTCTCGGAGGGCGAAGAATCTCGTGCTTTCAGC
TTCGATGTAGGAGGGCGTGGATATGTCCTGCGGGTAAATAGCTGCGCCGATGGTTTCTACAAAGATCGTTATGTTTATCGGCA
CTTTGCATCGGCCGCGCTCCCGATTCCGGAAGTGCTTGACATTGGGGAGTTCAGCGAGAGCCTGACCTATTGCATCTCCCGC
CGTGCACAGGGTGTCACGTTGCAAGACCTGCCTGAAACCGAACTGCCCGCTGTTCTCCAGCCGGTCGCGGAGGCCATGGAT
GCGATCGCTGCGGCCGATCTTAGCCAGACGAGCGGGTTCGGCCCATTCGGACCGCAAGGAATCGGTCAATACACTACATGG
CGTGATTTCATATGCGCGATTGCTGATCCCCATGTGTATCACTGGCAAACTGTGATGGACGACACCGTCAGTGCGTCCGTCGC
GCAGGCTCTCGATGAGCTGATGCTTTGGGCCGAGGACTGCCCCGAAGTCCGGCACCTCGTGCATGCGGATTTCGGCTCCAA
CAATGTCCTGACGGACAATGGCCGCATAACAGCGGTCATTGACTGGAGCGAGGCGATGTTCGGGGATTCCCAATACGAGGT
CGCCAACATCCTCTTCTGGAGGCCGTGGTTGGCTTGTATGGAGCAGCAGACGCGCTACTTCGAGCGGAGGCATCCGGAGCT
TGCAGGATCGCCGCGCCTCCGGGCGTATATGCTCCGCATTGGTCTTGACCAACTCTATCAGAGCTTGGTTGACGGCAATTTCG
ATGATGCAGCTTGGGCGCAGGGTCGATGCGACGCAATCGTCCGATCCGGAGCCGGGACTGTCGGGCGTACACAAATCGCCC
GCAGAAGCGCGGCCGTCTGGACCGATGGCTGTGTAGAAGTACTCGCCGATAGTGGAAACCGACGCCCCAGCACTCGTCCGA
GGGCAAAGGAATAGAGTAGATGCCGACCGGGAACCAGTTAACGTCGA**ATAACTTCGTATAATGTATGCTATACGAAGTTA**
TA

The sequences of promotor *PgdhA* and terminator *TtrpC* were respectively marked blue and red. The sequence underlined was multiple clone sites between promotor *PgdhA* and terminator *TtrpC*. The two loxP sequences was in in bold black. The hph expression cassette was marked with yellow.
